# Comment on Bažadona et al. The Interconnection between Carotid Intima–Media Thickness and Obesity: Anthropometric, Clinical and Biochemical Correlations. *Medicina* 2023, *59*, 1512

**DOI:** 10.3390/medicina60060981

**Published:** 2024-06-14

**Authors:** Christian Saleh

**Affiliations:** University of Basel, 4001 Basel, Switzerland; chs12us75010@yahoo.com

In a recently published paper in *Medicina*, Bažadona et al. reported on the relationship between carotid intima–media thickness (CIMT) and obesity [1]. The authors measured the CIMT according to the Mannheim consensus CIMT measurement protocol [2], namely in longitudinal view on the far wall of the common carotid artery (CCA), using an automated edge detection software. Bažadona et al. reported “that our study group, although obese and significantly older according to SCORE (Systematic COronary Risk Evaluation) had lower mean CIMT values compared with the reference values…When the CIMT of each patient was matched with its reference value, our adipose cohort had CIMT values as thin as the non-adipose, healthy population” [1]. The authors speculated as possible reasons for their findings “that we excluded patients with previous myocardial infarction, stroke, or TIA; additionally, hypertension and diabetes were diagnosed and treated in a timely manner so the impact of these risk factors on CIMT was smaller. The fact that the thickness of CIMT did not depend on the duration of hypertension and diabetes supports this explanation” [1].

There are some comments needed for a more exhaustive and balanced understanding of the results of this study.

## 1. CIMT Surrogate Marker of Atherosclerosis

CIMT is a surrogate marker of preclinical atherosclerosis [3,4,5]. It is noteworthy that atherosclerosis is mainly limited to the intima [6], while CIMT represents a composite ultrasound measure, encompassing the intimal and medial layers of the arterial wall [2,4]. Ultrasound imaging is not able to distinguish between intima and media [2,4]. An increase in the CIMT can have various reasons, such as media increase seen secondary to extracellular matrix glycosylation and the calcification of the media in diabetes or secondary to media hyperplasia in hypertension [7,8]. Carotid IMT increase can also be the consequence of intimal hyperplasia or intimal fibrocellular hypertrophy. However, these pathomophological substates are considered within an adaptive response of vascular remodeling and reflect an expression of an atherosclerotic process [7,9] with diabetes mellitus and arterial hypertension being cardiovascular risk factors for atherosclerosis [7,8,10,11,12].

## 2. Measurement Methods

There is no consensus as to the CIMT measurement protocol [13]. “The Mannheim carotid intima-media thickness and plaque Consensus paper” (henceforth called “Mannheim Consensus paper”) cited by Bažadona et al. [2] is not universally accepted. The last update of the “Mannheim Consensus paper” dates back to 2012 [2]. CIMT measurement as proposed here at a pre-established carotid section (i.e., the distal wall of the common carotid artery) [2] can coincide with a normal CIMT segment. It is important to bear in mind, however, that atherosclerosis, while systemic in nature, is asymmetric in presentation [14]. A single region measurement can miss therefore atherosclerotic altered vessel sections outside of the pre-established measurement area, resulting in misclassifying patients as normal. A composite CIMT measurement that includes both walls (i.e., proximal/distal) of the common carotid artery (CCA), the carotid bifurcation, and the internal carotid artery (ICA) [15] will provide a more accurate estimate of the actual CIMT and be a better predictor for cardiovascular disease than single-thickness measurements [16].

Bažadona et al. [1] measured only in one section, i.e., the distal wall of the CCA. A further critical aspect that Bažadona et al. [1] did not specify is if the CIMT measurement was synchronized with the cardiac cycle as proposed by the “Mannheim Consensus paper” [2]; CIMT varies during cardiac cycle, with CIMT reduction occurring during systole, given systolic lumen vessel expansion and CIMT increase occurring during diastole [17]. Therefore, the reduced CIMT reported by Bažadona et al. [1] could, if no synchronization occurred, be in part due to measurements occurring accidentally during the systolic cardiac phase.

## 3. Conclusions

The reason why, as indicated in Bažadona et al. [1], the “adipose cohort had CIMT values as thin as the non-adipose, healthy population” is very likely due to a suboptimal CIMT measurement protocol.

Therefore, to accurately assess subclinical atherosclerosis through an easily applicable and noninvasive ultrasonographic CIMT measurement, a consensus on the ultrasonographic measurement protocol still remains necessary [18].
